# Exploring Relationships between Recurrent Binge Eating and Illicit Substance Use in a Non-Clinical Sample of Women over Two Years

**DOI:** 10.3390/bs7030046

**Published:** 2017-07-18

**Authors:** Henry Kewen Lu, Haider Mannan, Phillipa Hay

**Affiliations:** 1 School of Medicine, Western Sydney University, Penrith NSW 2751, Australia; 17812812@student.westernsydney.edu.au (H.K.L.); H.Mannan@westernsydney.edu.au (H.M.); 2 Translational Health Research Institute (THRI), School of Medicine, Western Sydney University, Penrith NSW 2751, Australia

**Keywords:** recurrent binge eating, illicit substance use, binge eating disorder, longitudinal, co-morbidity, symptom trajectory

## Abstract

(1) Background: With the new edition of the Diagnostic and Statistical Manual of Mental disorders, 5th Edition (DSM-5), numerous parallels have been drawn between recurrent binge eating (RBE) and substance use disorders, with many authors examining RBE or binge eating disorder (BED) as a “food addiction”. The present study aims to clarify the relationship between recurrent binge eating (RBE) and illicit substance use (ISU) through investigating the temporal association between the two problems. (2) Methods: This study was embedded within a larger longitudinal study of non-clinical adult women recruited from Australian tertiary institutions. Participants responded at year 2 and year 4 of follow-up to the Eating Disorder Examination—Questionnaire. ISU was measured using a modified questionnaire taken from the Australian Longitudinal Study on Women’s Health. (3) Results: RBE and ISU co-morbidity was 5.88% in this non-clinical sample, and having one condition increased the likelihood of the other. The two conditions had a different trajectory over two years whereby ISU participants had significant risk of developing RBE in addition to or in place of their ISU but the reverse was not found for RBE participants. (4) Conclusion: This unidirectional relationship suggests that in spite of the similarities of RBE and ISU they may be distinct with respect to their co-morbidity over time.

## 1. Introduction

### 1.1. Background

Binge eating disorder (BED) is characterised by recurrent episodes of binge eating (RBE)—defined by an objective overconsumption of food and a sense of loss of control—without the compensatory behaviours which define bulimia nervosa. BED has an estimated lifetime prevalence between 1.9% and 2.8% depending on the population surveyed, making it the most common eating disorder [1,2,3]. It is categorised within the Feeding and Eating Disorder (ED) chapter of the Diagnostic and Statistical Manual of Mental disorders, Fifth Edition (DSM-5), and is thus distinct from substance use disorders (SUDs). However, parallels have been drawn between BED and SUDs by a number of authors, many of them examining BED as a “food addiction” [1,2,3,4]. Criterion A for SUD within the DSM-5 may be divided into groupings of “impaired control, social impairment, risky use and pharmacological criteria” [5]. These are comparable to the BED criteria of “a sense of a lack of control”, eating alone due to embarrassment, ongoing overeating despite negative consequences, and eating large amounts of food when not physically hungry. 

BED and SUD also share a number of psychological, neurobiological and genetic correlates. Factors such as neuroticism, impulsivity, sensation seeking and mood dysregulation are associated with both BED and SUD [6,7,8]. Animal models also support the theory that both BED and SUD follow from dysregulation of the same dopaminergic pathways [9,10] and have likewise been able to produce somatic withdrawal symptoms with sucrose cessation [11].

### 1.2. Co-Morbidity

Literature regarding the co-morbidity shared between RBE/BED and SUD supports the idea that there is an underlying shared pathology between the two conditions. In examining the literature, there are a number of classification issues leading to variability in reported rates of co-morbidity and prior to its recognition in the DSM-5 [5], BED was included as a type of Eating Disorders Not Otherwise Specified (EDNOS) [12]. Complicating matters further, numerous studies either have failed to specify the type of ED they had studied or have classified participants with inconsistent criteria [13]. For many studies RBE has been used to represent BED. For example, in a US national face-to-face survey of 9282 adults, 23% of those who had BED—which was defined as having 3 months or more of RBE—suffered also from a type of SUD [14]. The WHO World Mental Health Surveys support these findings, in which 23.7% of those with BED would have some form of SUD [15]. In exploring the prevalence of SUD, it is noted that the classification of substance abuse is similarly difficult; research in this field varies not only in the scope of substance abuse, ranging from a focus on a single substance to looking at SUDs collectively, but also the severity of substance abuse, studying one-time use as well as physiological dependence [13,16]. Harrop & Marlatt’s review [13] reflects these classification inconsistencies with co-morbidity prevalence ranging from 17–46% depending on ED and SUD types.

Illicit Substance Use (ISU) appears to be more common in ED populations than healthy controls; however, more information is needed to clarify the relationship between different types of illicit drugs and ED subgroups [16]. Cannabis [17,18] and opiate [17] use have found to be increased in those with an ED (subgroups combined) compared to controls. Evidence regarding amphetamine usage is inconsistent; one author reports associations of amphetamine usage with dieting and purging behaviour (without binging) [19,20] whilst another did not find increased use of amphetamines when comparing an ED group with the general population [17]. These findings may suggest that amphetamine usage may be associated with dieting and purging rather than with binging behaviour [16].

### 1.3. Longitudinal Predictors

On the other hand, longitudinal studies seem to suggest that however many similarities there may be between RBE/BED and ISU, they seem to differ in illness trajectories. A five-year longitudinal study of adolescent girls found that depressive symptoms were predictive of higher future levels of eating pathology and substance abuse (broadly defined and including alcohol use); eating pathology itself also predicted increased future substance abuse, with the inverse not being true [21]. Similarly, an Australian cohort study of adolescents and young adults found that even partial anorexia nervosa (AN)/bulimia nervosa (BN) diagnosis (where a participant satisfied two of four or three criteria for AN/BN) was predictive of amphetamine use [22]. The Growing Up Today Study has found that overeating (without a loss of control (LOC)) and RBE (overeating with a sense of a LOC) were both predictors for ISU; however, overeating alone was a stronger predictor for this outcome [23].

Fewer longitudinal papers have focused on predictors of RBE. Vogeltanze-Holm et al., found that the main factors predicting BED (strictly defined) was ISU in the past 12 months (odds ratio (OR) = 5.77, 95% CI = 1.64, 20.34) and more occasions of alcohol use until intoxication in the past 12 months (OR = 1.38, 95% CI = 1.03, 1.85) [24].

Finally, a five-year longitudinal study documenting the natural history of a variety of behavioural addictions over this period found a central effect of time on the problem behaviours, where the prevalence of the behaviours decreased and often resolved without intervention [25]. Excessive eating (examined over four years only) was found to decrease in prevalence at the same rate as comorbid substance use (broadly defined), with a mean 11.7% (SD = 2.3) suffering from comorbid SUD during the four-year time period.

These findings—in particular, that RBE/BED and ISU/SUD may not mutually predict risk for each other—suggest that perhaps distinct higher-order factors are mediating the relationship between RBE/BED and ISU/SUD rather than being controlled by the same underlying factor [13]. In a review of the phenomenology and treatment of behavioural addictions, Grant et al. hypothesises the opposite [26], claiming that one neurobiological dysfunction could give rise to multiple behavioural symptoms. The support for this theory comes from “consummatory cross-sensitisation” where prolonged intake and sensitisation with one substance can lead to increased consumption of another [4]. As a result of this cross-sensitisation, opiate- and stimulant-dependent individuals may have a cross-substitutability of preference for highly palatable foods, leading to reported cravings and binges [9,27].

Although there have been these studies of outcomes and putative symptoms substitution, it is notable that there have been few studies of the impact of comorbidity on other clinical features such as overall psychological distress, health-related quality of life and/or body weight, and findings have been mixed or inconsistent [28]. This may be of clinical importance if co-morbidity was found to be associated with poorer mental health and/or increased likelihood of obesity.

### 1.4. Aim and Hypotheses

In this study, we aimed to elucidate the nature of co-morbidity by (1) characterising the extent of the overlap of these two features within a non-clinical population, and (2) examining the trajectories of participants with regard to RBE and ISU over a period of two years’ time. We hypothesised that there would be significant co-morbidity between the two problems and, furthermore, that participants with RBE and those with ISU will have differing illness trajectories without mutual substitution between the two behaviours. We did not have specific hypotheses in regard to examining general psychological distress or health-related quality of life as these have been little studied in regard to the comorbidity of ISU and RBE.

## 2. Materials and Methods

### 2.1. Participants

Participants were 794 women initially recruited in 2004/2005 who were assessed repeatedly over a nine-year period (T0–T9). Any one follow-up assessment was not contingent on having competed any other follow-up. They were recruited through advertisements in four regional universities and vocational colleges (including adult students) in the Australian states of Queensland and Victoria for the purpose of a longitudinal study of community (non-clinical) women with and without eating disorder symptoms. Some participants were recruited via email and responded to the questionnaire online, whilst others were directly approached on campus locations and given hard-copy questionnaires and reply-paid envelopes. Due to these recruitment methods, characteristics of non-responders and overall response rate could not be measured.

**Figure 1:**
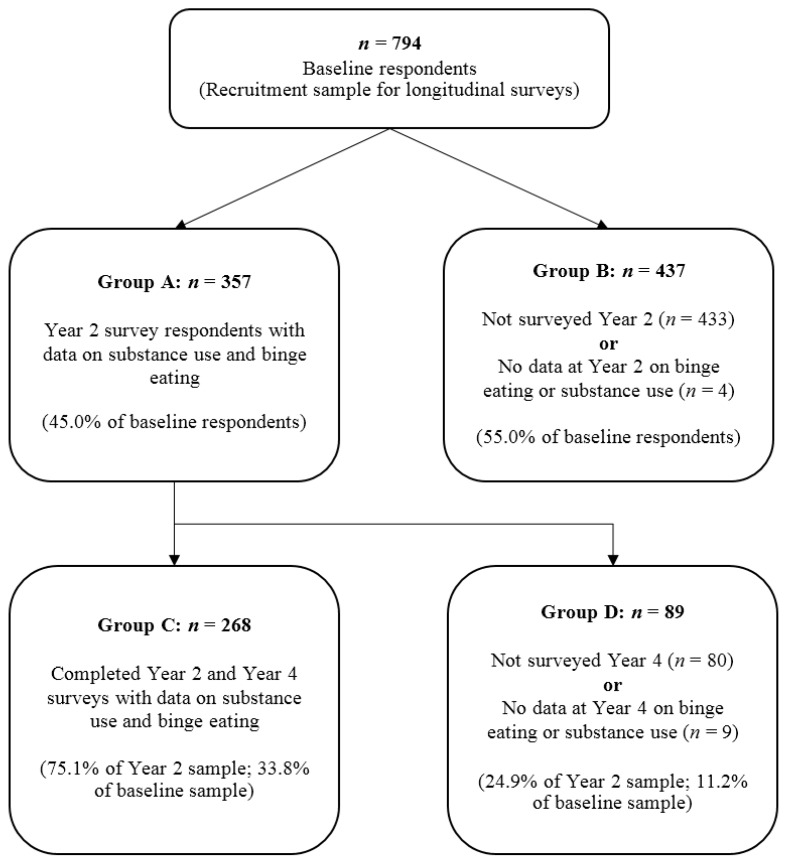
Participant flow from the beginning of the survey.

ISU was only assessed in T2 and T4 of the longitudinal study. As such, the present study comprises of participants who responded in T2 (*n* = 357) and those who responded in T4 who also responded in T2 (*n* = 268). Respondents who had no data for measures of binge eating or substance use in T2 (*n* = 4) or T4 (*n* = 9) were excluded. Figure 1 shows the participant flow through T1, T2 and T4 of the longitudinal study.

### 2.2. Procedures

The study was approved by the human research ethics committees (HREC) of the universities involved, with the Western Sydney University as lead HREC (Approval number 07/240). All participants completed written informed consent forms and there were no children requiring consent from a parent or guardian.

### 2.3. Measures

#### 2.3.1. Binge Eating

The Eating Disorder Examination Questionnaire (EDE-Q), a self-report questionnaire based on the Eating Disorder Examination (EDE) interview was used in order to assess eating disorder psychopathology. The EDE-Q has been validated in both community and clinical samples of patients with eating disorders and demonstrates close agreement with the EDE overall [29]. However, with regard to the complex features involved with binge eating behaviours the EDE-Q consistently generated higher levels of disturbance relative to the EDE [30].

Four items in the questionnaire targeted binge eating behaviours. The first two assessed objective binge eating (OBE), asking if the respondent had ever consumed what other people would regard as an unusually large amount of food, with a sensation of loss of control, and if so, how many times that had occurred over the past 28 days. This correlates with the DSM-5 criteria for recurrent binge eating episodes (Criterion A) but does not specify a discrete time period [5]. The second two assessed subjective binge eating (SBE), asking if the respondent had consumed a normal amount of food for the circumstances but had experienced a loss of control, and if yes, the number of times that had occurred. This is consistent with binge eating criteria being considered for the incoming International Classification of Diseases, 11th revision (ICD-11) [31], on the basis that people with subjective episodes have similar levels of impairment and distress related to binge eating as well as other psychopathology as do people with objective episodes [32,33]. As such, binge eating was coded as “present” if they had reported “yes” to either an objective or subjective binge for more than four episodes in the past 28 days and is used as the measure in this study of RBE. At the end of the EDE-Q there was a question asking current weight and height from which body mass index (BMI; kg/m^2^) scores were derived.

#### 2.3.2. Illicit Substance Use (ISU)

The questionnaire assessed frequency and amount of use of the following illicit substances: cannabis, amphetamines, hallucinogens, barbiturates, “ecstasy/designer drugs/cocaine”, “inhalants” and heroin. Participants were asked if they had used any of the illicit substances listed above in the past year, and if yes, the frequency of their current use over a one-month period. These questions were modified from the Australian Longitudinal Study on Women’s Health (ALSWH), where their frequency of use was categorised into scores of: 1 = less than monthly, 2 = monthly, 3 = weekly, 4 = two to three times per week, and 5 = daily [34]. The ordinal data gathered by this questionnaire allowed the creation of a new variable for the present study measuring overall ISU, which was calculated by taking the sum of the scores for each of the seven drug categories. A score of zero indicated no ISU, whilst the maximum score of thirty-five indicated that the participant was taking illicit drugs in all seven categories every single day of the week. The score is therefore influenced both by the range of illicit drugs consumed, as well as their frequency. For the purpose of this study, ISU was coded as present if the score was greater than 0, i.e., 1 or more.

#### 2.3.3. Psychological Distress

This was assessed with the Kessler-10 item distress scale (K-10) which was designed to detect cases of anxiety and affective disorders in the general population [35]. It is a 10-item instrument with an ordinal 5-point response to each question. It measures the level of distress and severity associated with psychological symptoms of depression and anxiety. The K10 is extensively used internationally, including in the WHO World Mental Health Survey and by government organizations in Australia, Spain, Colombia and Peru [36]. The advantages of the K-10 are its brief nature and its strong psychometric properties. It focuses on the previous 28 days thus is comparable in time-frame to the EDE-Q.

#### 2.3.4. Health-Related Quality of Life (HRQoL)

HRQoL was assessed with the well-validated 12-item Short Form-12 Health Status Questionnaire (SF-12) [37]. The SF-12 measures the impact of physical and mental ill-health on role limitations. It has been used extensively in research assessing impairment associated with physical and mental health conditions, and has robust psychometric properties, including in an Australian population sample [37,38]. It is a 12-item questionnaire that generates two weighted scales, a Physical Component Summary Scale (PCS) and a Mental Component Summary Scale (MCS), with each a mean of 50 and standard deviation of 10 in normative samples. Higher scores indicate higher levels of functioning.

### 2.4. Statistical Methods

Data were inspected for normality. Descriptive statistics were employed to report frequencies of socio-demographic variables, general symptoms, binge eating and substance use. Between-group differences were compared using ANOVA with post-hoc Tukey analyses for continuous normal data and Kruskal–Wallis and Mann–Whitney U tests for continuous non-normal data. The chi-squared test was utilised to test differences in distribution between categorical groups and ordinal data. Fisher’s exact test was utilised to calculate the p-value for the contingency tables given the small sample size. To determine whether a trajectory based on a transition from year 2 to year 4 was statistically significant, we tested the significance of estimated marginal probability for each trajectory based on the multinomial logistic regression of a multi-category outcome containing all possible combinations of RBE & ISU measured at year 4 conditional on the same outcome at year 2, while controlling for age and mental health-related quality of life both being measured at year 2. While assessing the relationship between a multi-category outcome containing all possible combinations of RBE & ISU measured at year 2 & the same outcome measured at year 4, both the control variables were found to be confounders. Listwise deletion of missing data was applied to the data at year 4 because the percentage missing at time 4 out of a total of 359 cases at time 2 who had any of the four possible RBE & ISU conditions was very low (3.06%), and hence complete case analysis would introduce very little bias. A significance level of *p* < 0.05 was employed for all tests. Analyses were conducted using IBM SPSS Statistics for Windows, version 22.

## 3. Results

### 3.1. Participant Features

Of the 357 participants who completed the follow-up survey at T2 (45.0% of baseline respondents), the median age (at T2) was 25 (Interquartile Range (IQR) = 15), 58.0% were unmarried or separated and 52.9% lived with family, friends or alone. The sample was well educated, with 33.9% achieving at least year 12, and 48.5% attaining a bachelor’s degree or higher. A large minority of the sample studied full-time (41.5%). Participants with symptoms were overrepresented in this study sample; compared to a previous general population study of Australian women, their Mental Health-Related Quality of Life scores (Short-Form 12 Mental Health Component Scores or SF-12 MCS) were lower and their EDE-Q subscale and global scores were higher, although lower than in clinical samples [39]. (See Section 2.3 for descriptors of these assessment measures.) ISU occurred in 20% (*n* = 72) of participants. Cannabis (*n* = 59, 82%) was the most frequently used substance followed by ecstasy/designer drugs/cocaine (*n* = 40, 56%). Other demographic and clinical features of the 357 participants at T2 can be found in Table 1.

Table 2 compares key characteristics of the four subgroups within the longitudinal study to assess if respondents were significantly different from non-respondents at T2 and T4. These are divided by response status and availability of RBE and ISU data. These groups are also outlined in Figure 1.

Year 2 respondents (Group A) were significantly older (MD = 2.23, SE = 0.74) than those who responded at baseline but were lost to follow-up (Group B), but were not significantly different in the other measures of body mass index (BMI (kg/m^2^)) and RBE characteristics. Participants who followed up at both year 2 and year 4 (Group C) were also significantly older (MD = 3.79, SE = 1.38) than their counterparts who did not respond in year 4 (Group D) and similarly, were not significantly different in BMI, RBE or ISU behaviours.

### 3.2. Co-Morbid ISU and RBE in the T2 Cohort

At T2, 226 of 357 (63.3%) respondents had neither RBE nor ISU behaviours; 55 (15.4%) had episodes of RBE *only*; the same number (*n* = 55, 15.4%) engaged in ISU *only*, whilst 21 participants (5.88%) in T2 admitted to engaging in both behaviours. 

The majority of participants who were identified as having a problem (either RBE or ISU) had one problem only and not the other (55/76, 72.4%) and this finding was not significant (χ^2^ = 2.32, df = 1; *p* = 0.09). As shown in Table 3 it was determined that those who had RBE had significantly higher frequency of ISU compared to those without RBE, and similarly, those who had ISU had significantly higher frequency of RBE compared to those without ISU.

Furthermore, participants with both ISU and RBE had the highest levels of eating disorder symptoms (global and subscale EDE-Q scores) and psychological distress (K-10 scores) and lowest levels of mental health HRQoL. These differences reached significance only for the findings of global EDE-Q scores compared to those with ISU alone, and K-10 scores compared to those with neither problem. Those with neither problem also had significantly lower EDE-Q global scores than all other groups and lower K-10 scores than those with RBE alone. These differences are shown in Table 4.

### 3.3. Participant Trajectories from T2 to T4

As shown in Table 5, the majority (*n* = 139, 82.2%) of participants with neither RBE nor ISU in T2 continued to have neither problem in T4, and 12% (*n* = 21) developed RBE. Almost half (*n* = 6, 46.2%) of those with both problems in T2 continued to have both problems in T4. Almost half (*n* = 18, 46.2%) of those with ISU in Year 2 continued to have ISU in year 4 and *n* = 7 (17.9%) developed an additional problem with RBE and *n* = 5 (12.8%) transitioned to RBE alone. The majority (*n* = 26, 57.8%) of those with RBE in T2 had neither problem in T4 and *n* = 17 (37.8%) continued to have RBE alone.

As shown in Table 6, participants with neither RBE nor ISU at year 2: were significantly more likely (*p* < 0.001) to remain that way or have RBE only by year 4, were significantly more likely (*p* < 0.01) to have ISU only by year 4, but were not significantly more likely to have both RBE and ISU by year 4. The most likely trajectory for those who were neither RBE nor ISU at year 2 was to remain that way by year 4. Participants being both RBE and ISU at year 2: were significantly more likely (*p* < 0.05) to have ISU only or both RBE & ISU by year 4, but were not significantly more likely to have RBE only or neither RBE nor ISU by year 4. The most likely trajectory for those who were both RBE and ISU at year 2 was to remain that way by year 4. Participants being RBE only at year 2 on the contrary: were significantly more likely (*p* < 0.05) to have RBE only or neither RBE nor ISU by year 4, but were not significantly more likely to have ISU only or both RBE & ISU by year 4. The most likely trajectory for those who were RBE only at year 2 was to have neither RBE nor ISU by year 4. For participants being ISU only at year 2 all transitions to year 4 were statistically significant with the most likely trajectory being both ISU only at year 2 and year 4.

## 4. Discussion

The current study investigated the relationship between RBE and ISU in a sample of Australian non-clinical adult women. The co-occurrence of ISU and RBE was examined cross-sectionally and then longitudinally over two years.

### 4.1. Comorbid Psychopathology

The hypothesis that RBE and ISU co-occur in the setting of a broader community sample was confirmed. Our study found that those with RBE had a higher frequency of ISU as well as the inverse, i.e., those with ISU had higher frequency of RBE. This co-morbidity might be explained by common neurobiological pathways involved in the two conditions [3,4] or may be a reflection of a self-mediated attempt at regulating negative affect [40], as demonstrated by Killeen et al., who found that past 30 day opiate use was correlated with increased EDE-Q scores [41]. Furthermore, our findings support those of Grilo et al. [42], which found that patients with BED with another concurrent psychiatric disorder had elevated levels of eating disorder psychopathology, although in this study this did not reach significance possibly because of small numbers of those with both problems.

### 4.2. Participant Trajectories and Between-Group Associations

Results from comparing participant numbers as they moved through from year 2 to year 4 demonstrated that participants with ISU were more likely to develop RBE either in addition to, or in place of their ISU, whereas those with RBE were likely to remain unchanged or spontaneously resolve over time, supporting our hypothesis that the two conditions take unique temporal courses and are differentially predictive for each other. Whist our findings are theoretically supportive of the existing literature in distinguishing RBE/BED and ISU/SUD, there are some differences in results. Measelle et al.’s [21] longitudinal study was similar in part to the present study and focussed on a variety of psychiatric disorders in adolescent girls and the temporal associations between symptom domains; in their study they established that there was a unidirectional relationship between BED and SUD—however, in their case pre-existing eating pathology predicted future growth in substance abuse but not the reverse—the opposite conclusion to this present study. This difference might be because of the shorter duration of this study, that Measelle et al. studied substance use more broadly and included alcohol abuse, and that we were studying subthreshold syndromes. Our findings however support the longitudinal findings of Vogeltanz-Holm et al. [24] that the main predictors for BED are ISU and alcohol intoxication. Furthermore, cessation of drug abuse followed by hyperphagia and weight gain is an established phenomenon in human studies [43] and animal models [44], although whether or not this disordered eating persists and develops into RBE/BED is a matter requiring further investigation.

### 4.3. Strengths and Limitations

The main strengths of this study include the reasonable sample size (*n* = 268) for the trajectory analysis and a 75.1% rate of retention of participants over the two-year follow-up period. However, the low numbers of those with both ISU and RBE may have limited finding statistical significance. The longitudinal design of the paper adds robustness to the findings presented in the study. However, the voluntary nature of recruitment and follow-up resulted in only 33.8% of baseline respondents being included for analysis, possibly contributing to elevated findings of eating disorder and ISU. Notably, we did not have a full assessment of the criteria for either BED or SUD, or more detailed assessment of RBE over a longer time frame, and did not assess for legal SUDs. Thus, we turned our focus to ISU and did not include legal substances such as alcohol and tobacco on the presumption that the act of breaching the law and risking the consequences of such more strongly implicates disordered substance use. Another important limitation is the non-inclusion of men as they have significantly higher rates of alcohol and drug use disorders; inclusion might produce altered co-morbidity rates and differing trajectories [45]. Further limitations include the single follow-up, self-report assessments of symptoms and BMI.

### 4.4. Clinical Implications

The key take-away from this study is that many participants with ISU went on to develop RBE, whilst those with RBE had a tendency for their behaviour to resolve over time. This is a relevant piece of information for clinicians in practice as it suggests that early assessment, monitoring and appropriate early intervention is important for individuals with ISU, and that this occurred despite the low threshold for defining ISU in this research. Finally, despite the stated differences between RBE and ISU, taking an addiction framework towards BED may improve current interventions or instigate the development of new treatments [8].

### 4.5. Future Directions

Studies investigating predictors for binge eating and its temporal associations are limited and, as such, further mixed-gender longitudinal studies conducted over longer periods of time would be warranted to clarify the associations between RBE and ISU. Other relevant aspects inviting possible future study include the investigation of alcohol or tobacco usage and binge eating behaviour as these commonplace drugs are also frequently consumed in excess. As a final point of interest, within the DSM-5, gambling and other behavioural addictions have been included within the same section as SUDs [46]. Given the co-morbidity between BED and SUD, it would be relevant to also consider the possible neurobiological and symptomatic correlates between full-threshold BED and other addictive disorders. It should be kept in mind as well that BED may be a construct distinct from the entity of “food addiction” which may present more similarities with SUD, and as such, further research is required in this area.

## 5. Conclusions

In this study, RBE and ISU have been found to be comorbid conditions in a non-clinical sample of young adult women, and furthermore, each condition increased the frequency of episodes of the other. Despite their similarities, the two conditions had a diverse trajectory over time, whereby ISU participants had higher likelihood of later developing RBE co-morbidly or in substitution but the reverse was not found for RBE participants. Further studies are indicated of full-spectrum BED and SUDs.

## Figures and Tables

**Table 1:** Descriptive data of 357 study participants in the present study.

	*n*	Mean	Standard Deviation	Median	Interquartile Range
Age/years at year 2	354	30.7	11.4	25	15
Body Mass Index (kg/m^2^)	342	24.8	5.5	23.5	5.3
Eating Disorder Examination—Questionnaire					
Weight concern subscale	353	2.04	1.54	1.80	2.60
Eating concern subscale	350	0.953	1.18	0.400	1.20
Shape concern subscale	346	2.37	1.58	2.13	2.63
Restraint subscale	352	1.57	1.43	1.20	2.00
Global Score	336	1.74	1.29	1.44	1.95
Illicit substance use frequency ^1^	357	0.65	1.72	0	0
Kessler 10 Psychological Distress Scale	351	17.5	6	16	7
Short Form-12 ^2^ Physical Component Score	348	52.1	8.2	54.5	8.3
Short Form-12 Mental Component Score	348	44.9	11.3	48.6	16.7

^1^ Illicit substance use frequency (range none to daily use) was a summed score of the seven drug categories. ^2^ The Short Form-12 is a measure of Health-Related Quality of Life.

**Table 2:** Participant characteristics of subgroups within the study.

Feature	Group A ^1^	Group B ^2^		Group C ^3^	Group D ^4^	
	Mean (SD) *n*		*t*, *p*	Mean (SD) *n*		*t*, *p*
Age	28.7 (11.4) *354*	26.5 (10.5) *426*	−2.85, 0.005	31.7 (12.02) *266*	27.9 (8.54) *88*	−2.74, 0.006
Body Mass Index (kg/m^2^)	24.3 (5.23) *336*	23.7 (5.52) *409*	−1.59, 0.11	24.91 (5.62) *254*	24.5 (5.17) *88*	−6.1, 0.545
	Median (IQR) *n*		*z*, *p*	Median (IQR) *n*		*z*, *p*
OBE/month	0 (0–1) *340*	0 (0–0) *408*	−3.34, 0.001	0 (0–0) *268*	0 (0–0) *89*	−0.62, 0.534
SBE/month	0 (0–2) *340*	0 (0–0) *408*	−2.75, 0.01	0 (0–2) *267*	0 (0–2) *89*	−0.09, 0.928
ISU	n.a. (ISU not assessed at baseline)			0 (0–0) *267*	0 (0–1) *89*	−1.70, 0.089

^1^ Participants who responded at T2 (with BE and ISU data), *n* = 357; ^2^ Participants who responded at baseline, but not at T2 *or* had no BE or ISU data at T2, *n* = 437; ^3^ Participants who responded at T2 *and* T4 (with BE and ISU data), *n* = 268; ^4^ Participants who responded at T2 but *not* at T4 *or* had no BE or ISU data at T4, *n* = 94; OBE = objective binge eating episodes; SBE = subjective binge eating episodes; ISU = illicit substance use; BE = binge eating; IQR = Interquartile Range.

**Table 3:** Comparative levels of illicit substance use (ISU) and recurrent binge eating (RBE) in participants with and without either problem.

Level of Behaviour	Median, IQ Range, *n*		Mann–Whitney U *Z, p*
*n = 357*	RBE	No RBE	
ISU	1, 0–6, *73*	0, 0–2, *282*	−2.612, 0.009
	ISU	No. ISU	
RBE	0, 0–6, *76*	0, 0–2, *281*	−2.234, 0.026

**Table 4:** Comparative clinical features of participants according to their RBE and ISU status.

	Neither ^1^	RBE ^2^	ISU ^3^	Both ^4^		Post-Hoc Tests with *p* < 0.05
Outcome	mean, SD, *n*				ANOVA F (df), *p*	Tukey test
EDE-Q Global	1.56, 1.21, 210	2.62, 1.38, 59	2.12, 1.43, 49	3.16, 1.23, 21	18.19 (3), <0.001	Neither ≠ Both, ISU, RBE; ISU ≠ Both
EDE-Q Restraint	1.17, 1.20, 219	2.59, 1.44, 59	1.46, 1.41, 50	2.87, 1.43, 23	27.33 (3), <0.001	Neither ≠ Both, RBE; ISU ≠ Both, RBE
EDE-Q Eating Concern	0.52, 0.74, 216	2.11, 1.29, 59	0.69, 0.73, 51	2.46,1.43, 23	70.62 (3), <0.001	Neither ≠ Both, RBE; ISU ≠ Both, RBE
EDE-Q Shape concern	1.86, 1.32, 210	3.82, 1.36, 59	2.01, 1.36, 53	3.89, 1.44, 23	44.24 (3), <0.001	Neither ≠ Both, RBE; ISU ≠ Both, RBE
EDE-Q Weight Concern	1.56, 1.28, 218	3.41, 1.42, 58	1.71, 1.24, 53	3.66, 1.43, 23	43.65 (3), <0.001	Neither ≠ Both, RBE; ISU ≠ Both, RBE
SF-12 MCS	45.94, 10.77, 219	43.04, 11.71, 59	44.31, 1.60, 52	41.09, 11.79, 23	2.13 (3), 0.096	n.a.
SF-12 PCS	52.61, 7.32, 291	51.24, 9.05, 59	53.15, 6.61, 52	51.20, 7.35, 23	0.89 (3), 0.45	n.a.
K-10 score	17.50, 6.23, 218	21.07, 7.65, 60	19.12, 7.8, 52	22.09, 7.99, 23	6.54 (3), <0.001	Neither ≠ RBE, Both
	median, IQ range, *n*				Kruskal–Wallis Χ^2^ (df), *p*	Mann–Whitney U Z, *p*
Body Mass Index (kg/m^2^)	23.6, 21.5–26.4 *211*	23.2, 20.8–29.2 *57*	23.5, 20.9–25.0 *50*	23.1, 20.2–25.0 *23*	1.924 (3), 0.588	n.a.

^1^ Participants with neither RBE nor ISU features; ^2^ Participants with RBE only; ^3^ Participants with ISU features only; ^4^ Participants with both RBE and ISU features; RBE = Recurrent Binge Eating; ISU = illicit substance use; EDE-Q = Eating Disorder Examination—Questionnaire, SF-12 = Short-Form 12; MCS/PCS = Mental health/physical health component score; K-10 = Kessler 10-item questionnaire.

**Table 5:** Longitudinal movement of participants between groups (*n* = 266).

	Year 4 Participants			
	*n* (%)			
Year 2 Participants	Neither	Both	ISU	RBE
Neither ^1^	139 (82.2)	2 (1.2)	7 (4.1)	21 (12.4)
Both ^2^	2 (15.4)	6 (46.2)	3 (23.1)	2 (15.4)
ISU ^3^	9 (23.1)	7 (17.9)	18 (46.2)	5 (12.8)
RBE ^4^	26 (57.8)	1 (2.2)	1 (2.2)	17 (37.8)

^1^ Participants with neither RBE n*or* ISU features; ^2^ Participants with both RBE and ISU features; ^3^ Participants with ISU features only; ^4^ Participants with RBE only; RBE = Recurrent Binge Eating ISU = illicit substance use.

**Table 6:** Estimated marginal probability with 95% confidence interval for each trajectory from year 2 to year 4 based on multinomial logistic regression controlling for age and mental health-related quality of life.

	Year 4 Outcome Estimated Marginal Probability with 95% Confidence Interval			
	Neither RBE nor ISU	Both RBE & ISU	RBE Only	ISU Only
**Year 2 Status**				
**Neither RBE nor ISU**	0.825 ^a^ (0.764, 0.886)	0.009 (−0.005, 0.024)	0.120 ^a^ (0.068, 0.172)	0.045 ^b^ (0.012, 0.078)
**Both RBE & ISU**	0.213 (−0.556, 0.481)	0.334 ^c^ (0.009, 0.659)	0.139 (−0.052, 0.331)	0.314 ^c^ (0.007, 0.621)
**RBE Only**	0.587 ^a^ (0.432, 0.741)	0.012 (−0.013, 0.036)	0.363 ^a^ (0.211, 0.515)	0.038 (−0.016, 0.095)
**ISU Only**	0.270 ^b^ (0.116, 0.425)	0.137 ^c^ (0.012, 0.263)	0.116 ^c^ (0.014, 0.217)	0.477 ^a^ (0.299, 0.654)

Note: a is *p* < 0.001, b is *p* < 0.01, c is *p* < 0.05 RBE = recurrent binge eating; ISU = illicit substance use.
